# The Burden of Protozoal and Helminth Intestinal Infections and Related Risk Factors Among the Households of Migrant Construction Workers in Bhopal

**DOI:** 10.7759/cureus.58413

**Published:** 2024-04-16

**Authors:** Akhila Reddy, Sagar Khadanga, Pradeep K Gupta, Karuna Tadepalli

**Affiliations:** 1 Preliminary Medicine, United Health Services, New York, USA; 2 Internal Medicine, All India Institute of Medical Sciences, Bhopal, IND; 3 Microbiology, Rajkiya Medical College, Jalaun, IND; 4 Microbiology, All India Institute of Medical Sciences, Bhopal, IND

**Keywords:** soil-transmitted helminths, protozoa, open field defecation, migrant workers, intestinal parasite, hand washing, construction workers

## Abstract

There are a few existing gaps and paucity of literature from Southeast Asia on the prevalence of intestinal parasitic infections among migrant construction workers. The present cross-sectional study was conducted to address this gap among migrant construction workers and their households in Bhopal. The study design included an interview questionnaire survey prior to the enrollment of participants and stool sample collection. The stool samples were processed according to the study protocol of macroscopy, occult blood testing, microscopy techniques combined with modified acid-fast, and sedimentation techniques. Participants were deemed positive if they exhibited microscopic findings in one out of three stool samples per recruit. We recommended clinical consultation for these cases and provided a report. Direct therapeutic intervention was not part of the study. The total recruits were 361. The predominant age group was young, i.e., aged 21 to 30 years (122/361, 33.8%), with the majority of females (55.2%). Most workers were occupied with work of digging soil (47.4%). The majority of participants (93.1%) practiced open field defecation (OFD). The prevalence of intestinal parasitic infections among migrant workers and households was 36.9% (133/361). Monoinfection was 88.7%, with 41% from *Entamoeba histolytica/Entamoeba dispar*.* *Monoinfection with *Hymenolepis nana* (10.2%)* *was a predominant helminth. The most common coinfection observed was of *Giardia intestinalis* with *Ancylostoma duodenale* (26.7%). Hand washing was the only independent predictor with an odds ratio of 3.6. Migrant behavior of the construction workers and their households was the major reason for not reaching the benefits of deworming schemes for children and vulnerability to intestinal parasitic infections.

## Introduction

Migrant workers make up almost half of the world's population, contributing remarkably to economic and social development [1]. According to the National Sample Survey Office (NSSO, 1999-2020), nearly 370 million workers constitute 92% of the total unorganized workforce of India [2]. The living conditions of workers and their families are poor, without basic amenities, exposing them to infections and health problems [3]. Different geographic regions have a varied spectrum of intestinal parasitic infections. One study from India documented an 81.2% prevalence of protozoan parasites compared to 18.8% helminths, contrasting studies from other parts of the world [4,5]. Prominent literature mentions *Giardia intestinalis *as prevalent and *Entamoeba histolytica* as the next protozoan. The most common cestode was *Hymenolepis nana *[6]. *Ascaris lumbricoides, Trichuris trichiura*, and *Ancylostoma duodenale *spectrum of soil-transmitted helminths were listed as the most prevalent [7]. A four-part strategy of the World Health Organization emphasizes the role of diagnosis of these gut parasites for the prevention and management of diarrheal diseases [8]. The microscopic examination of stool samples for morphological forms of parasites, such as larva, ova, trophozoite, and cysts, continues to be a gold standard laboratory diagnostic modality [9]. In the past, concentration techniques to improve diagnosis have been emphasized [10,11]. Other authors recommend screening three stool samples collected on alternate days for accurate diagnosis [12]. Two studies from Madhya Pradesh on the prevalence of intestinal parasites (VG Rao et al. (2001) in the Kundam block of the Jabalpur district and Tripathi K et al. (2013) in Bhopal) showed a prevalence of 59.5% and 40.7%, respectively [13,14]. *G. intestinalis *was the most common protozoan at 43%, and *Ascaris* *lumbricoides w*as a prevalent helminth (9.8%). The quality of drinking water depended on cloth filtration (49%) and plain water (41.7%) consumption in rural settings; while in urban settings, 34.8% consumed plain municipally supplied water while 15% consumed post-filtration water. Open field defecation (OFD) among children in rural settings was 7.3% while 3% from urban localities. Untrimmed nails and poor hygiene in 86% of rural and 28.7% of urban school-going wards were documented [14]. Intestinal parasitic infection is singly and endemically, worldwide, the greatest contributor to morbidity and disease. Several factors, such as the hot humid climate of the tropics, limited access to potable water, poverty, illiteracy, compromised living conditions, and hygiene, are associated with these infections. These intestinal parasitic infections constitute a global health burden leading to 450 million clinical morbidities predominately in mostly reproductive-age women and children from developing nations [15]. *Ascaris lumbricoides*, *Trichuris trichiura*, *A. duodenale*, and *Necator americanus* are soil-transmitted helminths documented by the WHO in 2012, to affect more than 2 billion population across the world; nearly 1 billion with *Ascaris* *lumbricoides *itself. Considering poor literature evidence on the spectrum and prevalence of intestinal parasitic infections among migrant construction workers and their households from the state of Madhya Pradesh, this study was undertaken. The specific objectives of this study were to study the prevalence of intestinal parasitic infections among migrant construction workers and their families to determine the spectrum of intestinal parasitic infections in relation to age, nature of work, gender, and single or multiple intestinal parasitic infections, and to assess major associated risk factors that predispose construction workers and their households to intestinal parasitic infections.

The study manuscript was initially posted to the https://www.preprints.org/ preprint server on 29 December 2023 [16].

## Materials and methods

Study site and design

All India Institute of Medical Sciences Bhopal (AIIMS Bhopal) in Madhya Pradesh, Central India, a tertiary care medical institute of national importance, had significant migrant construction workers placed at its site during its construction phase. Hence, a prevalence study of intestinal parasitic infections was conducted for this population.

Study setting and study population

The study was done in the Department of Microbiology, AIIMS Bhopal from February 2016 to September 2016. Households of migrant construction workers, including the workers themselves, working at AIIMS Bhopal, both adults and children aged more than six months, were the study participants.

Sample size and sampling criteria

The sample size for this study was 384, considering an expected prevalence of 50% (maximum value), because of the absence of recent data on the prevalence of intestinal parasites among migrant construction workers, specifically from the central region. Inclusion criteria involved the ability to give informed consent and workers or households who have not received any anti-parasitic treatment in the last three months. Those not responding to the questionnaire or responding only to the questionnaire without submitting stool samples for testing were excluded from the study.

Data collection and analysis

A semi-structured interview questionnaire was prepared first in English and then translated to Hindi to gather demographic and risk factor information by asking questions on demographic data (i.e., age, gender, and education level), socioeconomic background (i.e., nature of work, household income, and educational status), behavioral risks (i.e., personal hygiene, such as hand washing and food consumption), environmental sanitation and living condition characteristics (i.e., types of water supply and latrine system), and health conditions with history of symptoms (i.e., diarrhea, nausea, vomiting, and abdominal pain). For children (less than 12 years old), answers from the questionnaire were double-confirmed via interviews with their parents or guardians who had given informed consent. The interview questionnaire survey was in the local language. This was followed by instructions to workers on appropriate stool sample collection and transportation to the laboratory. For each participant, it was done prior to enrollment and receiving stool samples. For the collection of stool samples, each participant was given three universal screw-capped plastic bottles with a wooden scoop and instructed to fill it to the halfway mark on the bottle and for the scoop to be discarded after use. To reduce inappropriate samples, they were instructed to refrain from taking any oily emulsions, barium salts, or bismuth medications. Each participant was requested to collect stool samples on alternate days so three samples were collected from each participant. The samples were transported immediately to the microbiology laboratory at AIIMS Bhopal. Immediately upon receipt, the laboratory performed a macroscopic examination and occult blood test, followed by microscopy techniques, such as wet mount with saline and iodine. On suspicion of coccidian parasites by wet mount, modified acid-fast staining was performed. Leftover samples were processed simultaneously for sedimentation and floatation techniques. The remaining samples were preserved with 10% formalin till the study duration. Preservation of fecal samples was necessary to retain protozoal morphology and to avoid further morphological development of helminth eggs and larvae. If processing was delayed, the samples were refrigerated at 4°C. Recruits who were diagnosed with pathogenic protozoa or helminth parasites in one of the three stool samples sent were marked as positive and a report was dispatched to the recruits by the laboratory for clinical consultation. Laboratory parasitological examination procedures included the macroscopic examination of stool samples. The collected stool samples were physically examined for properties, such as color, consistency (formed, loose, or watery), and presence of blood, mucus, and worms. All stool samples were tested for fecal occult blood by commercial Cancheck-FOBT Rapid test (Tulip Diagnostics, Goa, India). Then, a direct wet mount with normal saline (0.85% NaCl solution) was prepared and observed for the presence of motile intestinal parasitic forms, such as larva of helminths, trophozoites of protozoan intestinal parasites, and helminthic eggs, under light microscope. Lugol's iodine staining (1%) was used to observe cysts of protozoan intestinal parasites. For the floatation method, the saturated salt solution method was performed. Roughly 1 gm of stool sample was taken in a 30 ml glass beaker and mixed with a few drops of saturated saline solution (SSS). By stirring continuously, a suspension was made. SSS was added to fill the container. The crude matter that floated on top was removed. The beaker was placed on a level surface and the final filling of the beaker was done until a convex meniscus was formed. Then, a plain glass slide was carefully placed on the top of the beaker, with the central portion of the slide touching the fluid. It was then allowed to stand for nearly 20-30 minutes. Then, the glass slide was swiftly lifted and gently turned to prevent spillage of the liquid. A coverslip was placed over the fluid on the slide. The prepared wet mount was microscopically examined. The formol-ether concentration technique was also performed as a sedimentation method. A half teaspoon of stool sample (approximately 1 gm) was added to a centrifuge tube containing 10 ml of 10% formalin and allowed to stand for 30 minutes. Then the fecal suspension was filtered through double-layered gauze and funneled into a 15 ml centrifuge tube. Saline (85%) was added to the centrifuge tube to bring the fluid level within several millimeters of the rim of the tube. The tube was centrifuged at 500 g for 10 minutes. The supernatant was then discarded, and the sediment was resuspended in saline, nearly filling up to the brim of the tube. The tube was centrifuged again for 10 minutes at 500 g. The supernatant was discarded and the sediment resuspended in 7 ml of 10% formalin and 3 ml of diethyl ether. The tube was closed with a stopper and shaken well for seconds; after which, it was centrifuged at 500 g for 10 minutes, and then allowed to stand for five minutes. Four layers were formed: a sediment layer at the bottom of the tube containing protozoal cysts and helminth eggs, a formal saline layer, fecal debris on top of the formal saline layer, and a topmost layer of ether. The plug of fecal debris was removed by piercing around it with an applicator stick, taking care not to disrupt the debris. All fluid was then discarded into the discarding jar with a firm swing, leaving behind one or two drops of fluid with sediment. Sediment was mixed with an applicator stick, and a trace amount was examined as normal saline and iodine wet mounts under the light microscope with low-power and high-power objectives. A modified Ziehl-Neelsen staining to detect oocysts of coccidian intestinal parasites was done for those stool samples on the saline wet mount where the oocysts were suspected. First, thin smears directly from fresh stools and from sediments of concentrated stools were prepared and allowed to air dry. Then the slides were fixed with methanol for two to three minutes. A control slide was included with each batch of staining. Fecal samples were stained with cold strong commercial carbol fuchsin (Himedia) for 10 minutes. After washing the slides in tap water, they were decolorized with 1% HCL in 95% ethanol until color ceased to flood out, rinsed in tap water, and followed by counterstaining in methylene blue for 30 seconds. Then, the slide was rinsed in tap water and allowed to dry. Finally, the slides were observed under a light microscope with a low-power magnification to detect the oocysts and oil immersion with the objective of identifying the oocysts.

Statistical analysis

The operational dependent variable was infection and the independent variables were age, occupation, family income, family size, source of water and its handling, availability of latrines, personal hygiene, good personal hygiene, hand washing practices, nail trimming, use of footwear, and consumption of vegetables, fruits, and meats. Collected data were coded, checked, and analyzed using SPSS version 16.0 (SPSS Inc., Chicago, IL). Descriptive statistics, such as frequency, percentage, mean and standard deviation, and range, were determined for each intestinal parasite. To test the null hypothesis, inferential statistical analysis of comparisons between two categorical variables was carried out using the chi-square (x2) test to verify the relationship between independent factors and the outcome variables. Logistic regression was used to determine the strength of the association between infection prevalence and potential risk factors using odds ratio (OR) and 95% confidence interval (CI).

Ethical considerations

Approval from the Institutional Human Ethics Committee (IHEC), AIIMS Bhopal (LOP/2016/STS0078) for carrying out the study was obtained. Written informed consent forms in English and Hindi were prepared to obtain consent from participants, along with a participant information sheet. All the participants, both adults and children, in whom intestinal parasitic infection was detected were recommended treatment with the appropriate drug and dosage by dispatching official reports and clinical consultation.

## Results

A total of 389 migrant workers, including their households, working at AIIMS Bhopal consented to participate in this study. Out of 389 participants, 28 were excluded from the study, as they did not participate in the questionnaire survey and did not return with their stool samples for the study.

From 361 participants, 33.88% were in the age group of 21 to 30 years, 55.2% were females, 62.3% received primary school education, 47.4% were occupied with daily work of digging soil and carrying mud, 85.6% earned >Rs. 3000 and <Rs. 5000, and 97.8% were with a family size of < five members/family. For 98.6% of the participants, the water source was the municipal tap water supply, and 96.2% consumed water directly without prior filtering or boiling. Of the participants, 71.2% had unclean and untrimmed nails, 74% consumed unwashed raw or cooked vegetables and fruits, 8% never wore footwear, and 3% did not wash their hands prior to eating. Of the participants, 93.1% practiced open-field defecation (Tables 1, 2). These parameters are detailed further based on positive or negative results for intestinal parasitic infections (Tables 3-5).

**Table 1:** Socio-demographic characteristics of the study participants

Characteristics		Total No. (n = 361)	Percent (%)
Age	6 months to 10 years	65	18
	11 years to 20 years	79	21.9
	21 years to 30 years	122	33.8
	31 years to 45 years	70	19.4
	46 years to 60 years	18	5
	>60 years	7	1.9
Gender	Male	159	44
	Female	201	55.2
	Transgender	1	0.3
Educational status	Illiterate	123	34.1
	Primary school	225	62.3
	Secondary school and above	13	3.6
Occupation	Mason	20	5.5
	Plasterer	25	6.9
	Heavy machine operator	27	7.5
	Plumber	14	3.9
	Metal worker and welder	19	5.3
	Carpenter	05	1.4
	Digs soil and carries mud	171	47.4
	Student	35	9.7
	No work	45	12.5
Family income	<3000 Rs./month	3	0.9
	>3000 Rs./month and <5000 Rs./month	309	85.6
	>5000 Rs./month and <10000 Rs./month	49	13.6
Family size	<5	353	97.8
	>5	8	2.2

**Table 2:** Drinking water source, type, and personal hygiene of the participants

Characteristics		Total No. (n = 361)	Percent (%)
Water source	Municipal tap	356	98.6
	Private tap	3	0.9
	Well	2	0.6
	Pond	0	0
Drinking water	Filtered	0	0
	Boiled	12	3.3
	Direct consumption	349	96.2
Personal hygiene	Good	93	25.8
	Careless	268	74.2
Type of toilet used	Private toilet	0	0
	Public toilet	22	6.1
	Open field defecation	339	93.1
Hand washing	Eating without handwashing	11	3
	Both after defecation and before meals	350	97
Vegetables and fruits consumed	Washed and eaten cooked or raw	1	0.3
	Unwashed and eaten cooked or raw	267	74.0
	Only cooked vegetables consumed	93	25.8
Meat consumption	Uncooked	0	0
	Partially cooked	0	0
	Properly cooked	110	30.5
	Do not consume	251	69.5
Footwear	Always	193	53.5
	Never	29	8.0
	Occasionally use	139	38.5
Trimmed and cleaned nails	Yes	104	28.8
	No	257	71.2

**Table 3:** Distribution of respondents based on intestinal parasite findings (n = 361)

Participants	Total numbers (n = 361)	Percentage (%)
Positive for intestinal parasitic infection	133	36.9
Negative for intestinal parasitic infection	228	63.7
Monoinfection	118	32.7
Co-infections	15	4.2

**Table 4:** Prevalence of intestinal parasitic infections based on characteristics among migrant construction workers and their households

Factors	Categories	Infection		Total (n = 361)
		Negative (n = 228), %	Positive (n = 133), %	
Age	6 months to 10 years	(45) 19.7%	(20) 15%	(65) 18.0%
	11 years to 20 years	(50) 21.9%	(29) 21.8%	(79) 21.9%
	21 years to 30 years	(76) 33.3%	(46) 34.6%	(122) 33.8%
	31 years to 45years	(44) 19.3%	(26) 19.5%	(70) 19.4%
	46 years to 60 years	(9) 3.9%	(9) 6.8%	(18) 5.0%
	>60 years	(4) 1.8%	(3) 2.3%	(7) 1.9%
Gender	Male	(96) 42.1%	(63) 47.4%	(159) 44.0%
	Female	(131) 57.5%	(70) 52.6%	(201) 55.7%
	Transgender	(1) 0.4%	(0) 0.0%	(1) 0.3%
Educational status	Illiterate	(68) 29.8%	(55) 41.4%	(123) 34.1%
	Primary school	(150) 65.8%	(75) 56.4%	(225) 62.3%
	Secondary school and above	(10) 4.4%	(3) 2.3%	(13) 3.6%
Nature of work	Mason	(11) 4.8%	(9) 6.8%	(20) 5.5%
	Plasterer	(17) 7.5%	(8) 6.0%	(25) 6.9%
	Heavy machine operator	(14) 6.1%	(13) 9.8%	(27) 7.5%
	Plumber	(10) 4.4%	(4) 3.0%	(14) 3.9%
	Metal worker and welder	(9) 3.9%	(10) 7.5%	(19) 5.3%
	Carpenter	(4) 1.8%	(1) 0.8%	(5) 1.4%
	Digs soil and carries mud	(110) 48.2%	(61) 45.9%	(171) 47.4%
	Student	(25) 11.0%	(10) 7.5%	(35) 9.7%
	No work	(28) 12.3%	(17) 12.8%	(45) 12.5%
Family income	<3000 Rs./month	(2) 0.9%	(1) 0.8%	(3) 0.8%
	>3000 Rs. and <5000 Rs./month	(198) 86.8%	(111) 83.5%	(309) 85.6%
	>5000 Rs. and <10,000 Rs./month	(28) 12.3%	(21) 15.8%	(49) 13.6%
Family size	<5	(224) 98.2%	(129) 97.0%	(353) 97.8%
	>5	(4) 1.8%	(4) 3.0%	(8) 2.2%

**Table 5:** Prevalence of intestinal parasitic infections based on drinking water source, type, and personal hygiene of the migrant workers and their households

Factors	Categories	Infection		Total (n = 361)
		Negative (n = 228), %	Positive (n = 133), %	
Water source	Municipal tap	(225) 98.7%	(131) 98.5%	(356) 98.6%
	Private tap	(2) 0.9%	(1) 0.8%	(3) 0.8%
	Well	(1) 0.4%	(1) 0.8%	(2) 0.6%
Type of drinking water	Boiled	(10) 4.4%	(2) 1.5%	(12) 3.3%
	Direct consumption	(218) 95.6%	(131) 98.5%	(349) 96.7%
General personal hygiene	Good	(65) 28.5%	(28) 21.1%	(93) 25.8%
	Careless			
		(163) 71.5%	(105) 78.9%	(268) 74.2%
Type of toilet used	Public toilet	(14) 6.1%	(8) 6.0%	(22) 6.1%
	Open field defecation	(214) 93.9%	(125) 94.0%	(339) 93.9%
Hand washing	Eating without hand washing	(4) 1.8%	(7) 5.3%	(11) 3.0%
	Both after defecation and before meals	(224) 98.2%	(126) 94.7%	(350) 97.0%
Vegetables and fruits	Washed and eaten cooked or raw	(0) 0.0%	(1) 0.8%	(1) 0.3%
	Unwashed and eaten cooked or raw	(171) 75.0%	(96) 72.2%	(267) 74.0%
	Only cooked vegetables consumed	(57) 25%	(36) 27.1%	(93) 25.8%
Meat	Properly cooked	(68) 29.8%	(42) 32.6%	(110) 30.5%
	Do not consume meat	(160) 70.2%	(91) 68.4%	(251) 69.5%
Wearing shoes	Always	(122) 53.5%	(71) 53.4%	(193) 53.5%
	Never	(20) 8.8%	(9) 6.8%	(29) 8.0%
	Occasionally use	(86) 37.7%	(53) 39.8%	(139) 38.5%
Trimmed and cleaned fingernails	Yes	(69) 30.3%	(35) 26.3%	(104) 28.8%
	No	(159) 69.7%	(98) 73.7%	(257) 71.2%

Out of 361 respondents, 36.9% showed intestinal parasitic infection. Out of 133 participants with positive intestinal parasite findings, 88.72% had monoinfection and 11.28 % had co-infections with more than one parasitic infection (Table 3).

**Figure 1:**
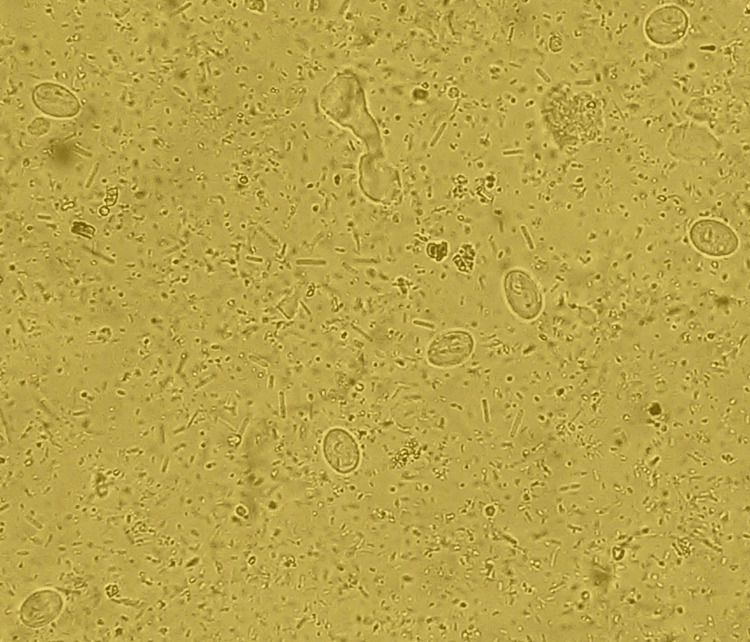
Giardia intestinalis cysts (100x magnification), iodine wet mount

**Figure 2:**
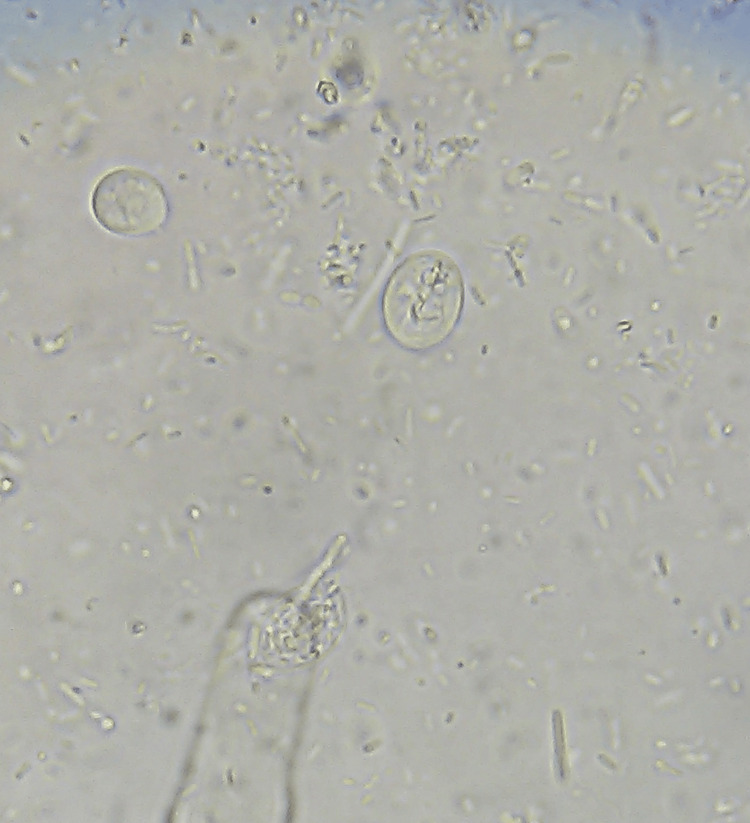
Giardia intestinalis cyst (400x magnification), saline wet mount

**Figure 3:**
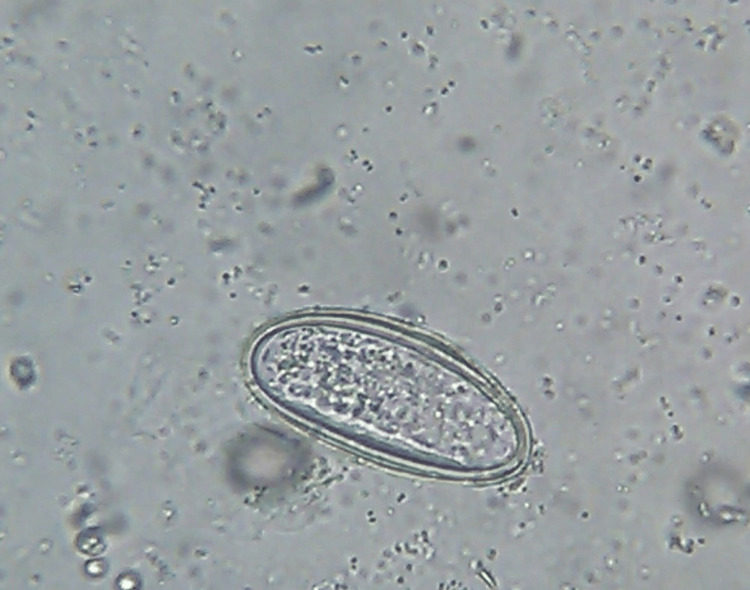
Plano-convex non-bile-stained egg of Enterobius vermicularis (400x magnification), saline wet mount

**Figure 4:**
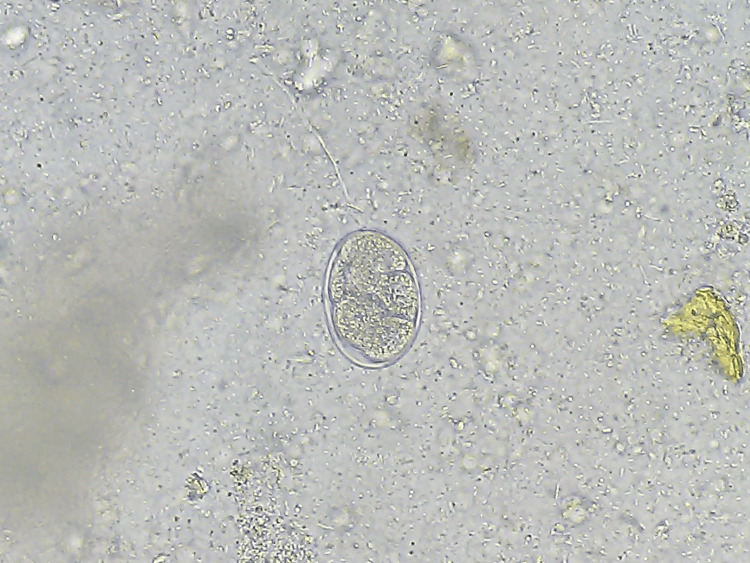
Non-bile-stained egg of Ancylostoma duodenale (400x magnification), saline wet mount

**Figure 5:**
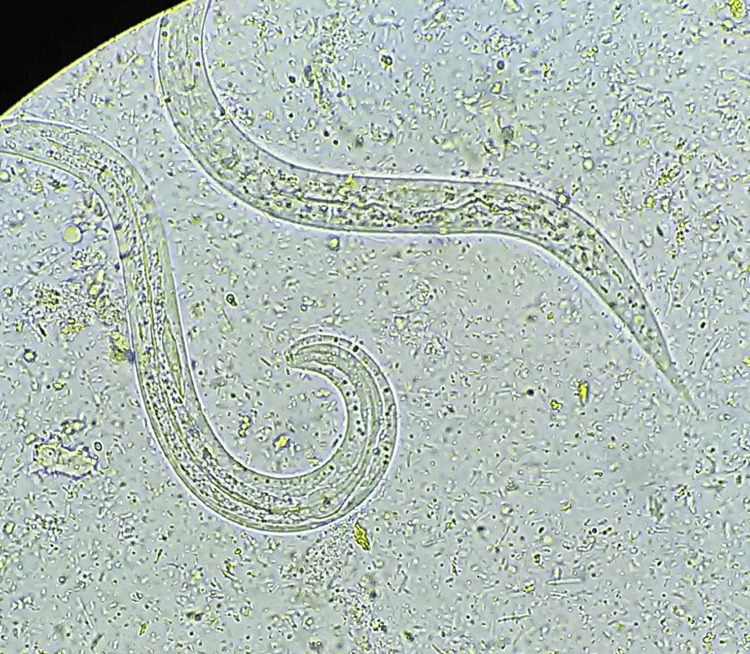
Larva Strongyloides

**Figure 6:**
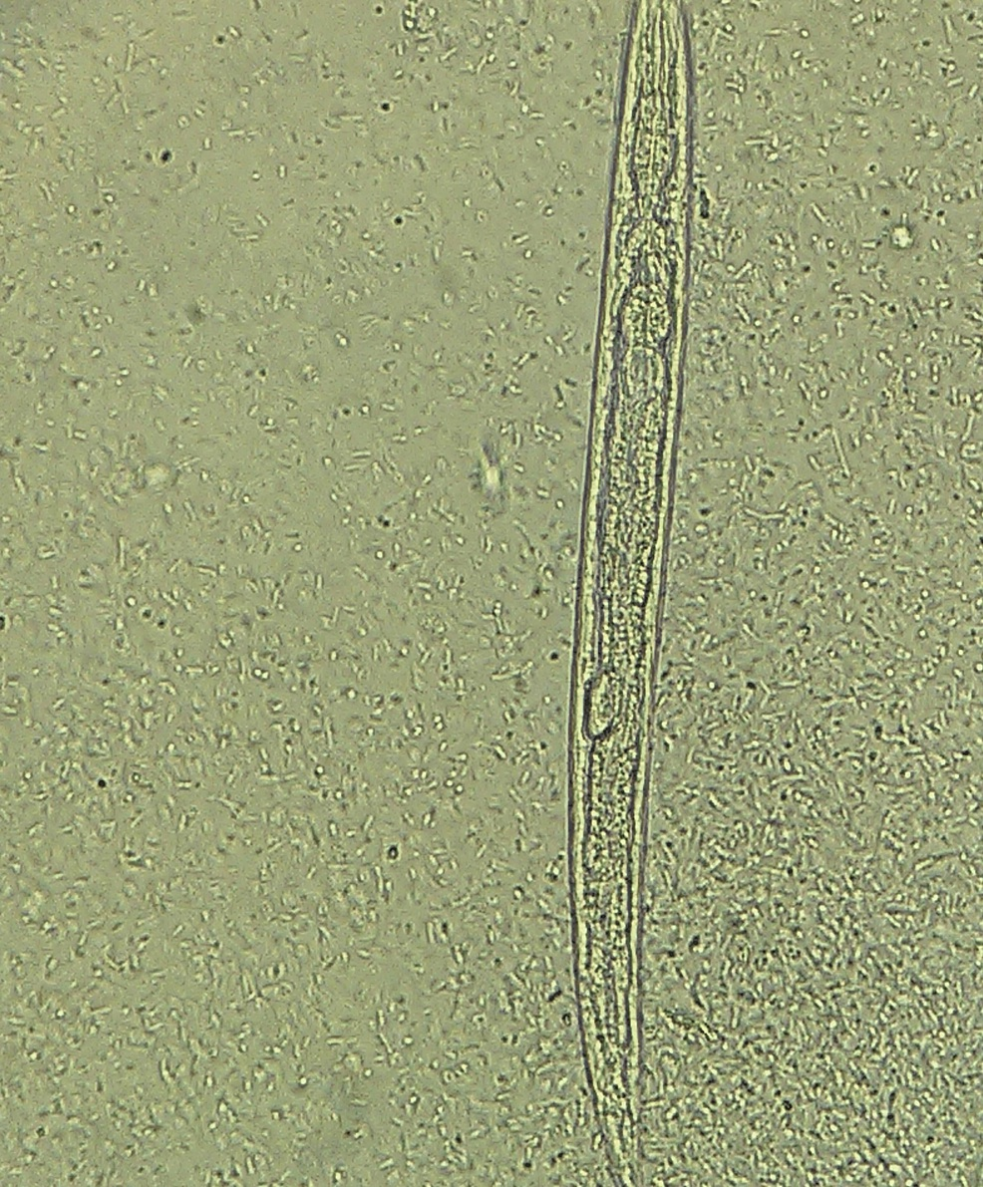
Strongyloides species rhabditiform larva (400x magnification), saline mount

The spectrum of monoinfection and co-infection with intestinal parasites was analyzed. The most common parasite found among positive monoinfected respondents was the protozoan parasite *E. histolytica/E.* *dispar*, followed by *Giardia intestinalis*. Among the helminths, *Hymenolepis nana* was the most common parasite causing monoinfection with *Strongyloides *species as the second commonest helminth causing monoinfection (Table 6 and Figures 1-6).

**Table 6:** Distribution of intestinal parasites among monoinfected participants (n = 118)

Parasites found	Total number	Percent (%)
Entamoeba histolytica/dispar	48	40.67
Giardia intestinalis	37	31.45
Hymenolepis nana	12	10.17
Strongyloides spp.	10	8.47
Ancylostoma duodenale	9	7.62
Enterobius vermicularis	2	1.69

The most common co-infection of parasites among respondents was *Giardia intestinalis* with *Ancylostoma duodenale* (Table 7).

**Table 7:** Distribution of intestinal parasites among co-infected participants (n = 15)

Parasites found	Total number	Percent (%)
*E. histolytica* + *G. intestinalis*	3	20
*E. histolytica* + *H. nana*	2	13.3
*E. histolytica* + *A. duodenale*	3	20
*G. intestinalis* + *H. nana*	3	20
*G. intestinalis* + *A. duodenale*	4	26.7

Results of assessment of associated risk factors

We used logistic regression analysis to identify independent predictors of infection. For this, we have considered infection as a dependent variable, which was coded as 1 for positive (presence of intestinal parasitic infection) and 2 for negative (absence of any intestinal parasitic infection) (Table 8).

**Table 8:** Chi-square (x2) test to verify the relationship between independent factors and the outcome variables

Variables	Chi-square	df	P-value
Educational status	4.570	2	0.102
Nature of work	6.293	8	0.614
Water source	0.180	2	0.914
Type of drinking water	2.016	1	0.156
General personal hygiene	1.895	1	0.169
Type of latrine used	0.001	1	0.972
Hand washing	3.758	1	0.053
Vegetables and fruits	2.232	2	0.328
Meat	0.324	1	0.570
Wearing shoes	0.633	2	0.729
Trimmed and cleaned fingernails	0.698	1	0.403

For the identification of independent variables, we have used a data-driven approach where variables in univariate analysis with p = 0.25 were included as independent factors. These variables were educational status, hand washing before eating, and source of drinking water. The omnibus test of model coefficients was statistically significant (p = 0.032), which indicates that the model is significant. Hosmer and Lemeshow's test was not significant (p = 0.714), which indicates that the data fit the model.

Table 9 shows the odds ratios and their confidence intervals through logistic regression. It can be seen from the table that only handwashing was an independent predictor having an OR of 3.6 with a CI of 1.00-13.00, indicating that those who do not wash their hands before meals are 3.6 times more likely to develop infection.

**Table 9:** Analysis of risk factors associated with intestinal infections using logistic regression SE: standard error.

Variables		B	SE	Wald	df	P-value	OR	95% CI for OR	
								Lower	Upper
Step 1a	Education			4.305	2	0.116			
	Education (1)	0.791	0.691	1.311	1	0.252	2.206	0.569	8.545
	Education (2)	0.341	0.682	0.25	1	0.617	1.407	0.37	5.35
	Drinking water (1)	-1.097	0.802	1.872	1	0.171	0.334	0.069	1.608
	Hand washing (1)	1.286	0.654	3.872	1	0.049	3.618	1.005	13.026
	Constant	-1.077	0.667	2.604	1	0.107	0.341		
Variable(s) entered on step 1: Education, drinking water, and hand washing.									

## Discussion

Studies have mentioned the recent efforts at providing deworming, toilet facilities, and hand washing awareness among the general public and schools. These facilitate as preventative measures for reducing the burden of intestinal parasitic infections.

There have been very few studies addressing the migrant construction workers who differ from agricultural workers, as migrant workers and their families do not stay in one place. Due to their nomadic behavior and work requirements, they are usually very susceptible to intestinal parasitic infections and incidentally, it is difficult to include them in the deworming programs for children at schools. Nonspecific symptoms such as weakness and abdominal pain justify the observation and screening for intestinal parasites among construction workers and their families as they are the most vulnerable. The present study comprised 361 participants, out of which 133 were positive for one or more intestinal parasitic infections and 228 were negative. This study shows the prevalence of intestinal parasitic infections among migrant construction workers and their households as 36.9%. Prevalence, specifically among Indian construction workers and their households, is lacking, but the overall prevalence reported from various studies is similar. Tripathi et al. showed a prevalence of 40.7% in their study [14]. Studies from northern parts of India, in low socioeconomic areas of Chandigarh, had nearly 19.3%. Urban and rural regions of Kashmir valley documented at least one of the helminths in 71.2% of the enrolled population with 68% *Ascaris lumbricoides*, followed by *Trichuris trichiura* (28%), *Enterobius vermicularis* (13%), and cestode *Taenia saginata* (5%) [17,18]. Studies have emphasized the need for routine monitoring of public health related to intestinal parasitic infections across varied populations [4]. These parasites reside in the gut of humans and animals. Urbanized parts of the world reported more protozoan intestinal parasites compared to helminths. Amoebiasis is still the third most important cause of mortality in developing countries. The WHO estimates that nearly 50 million suffer insidious amoebic infections annually, leading to 40,000-100,000 deaths annually. Previously documented were over a billion *A. lumbricoides*, almost 795 million *T. trichiura,* and nearly 740 million infected by *A. duodenale* infections worldwide [19]. Intestinal helminths lead to chronicity of illness and nutrition leading to the stunted physical and mental growth of children. This impairs their education and livelihood. Studies from African countries have documented *A. lumbricoides, Necator americanus, T. trichiura*,* Strongyloides stercoralis, E.* *histolytica, *and *G. intestinalis* as prevalent gut parasites, attributing these infections to deprived socioeconomic conditions and lack of awareness. A recent East African meta-analysis documented a pooled prevalence of 38.54% intestinal parasitic infections in pregnant women. Statistically significant variables associated with the burden were rural location, toilet facility, consumption of uncooked fruits and vegetables, and unprotected drinking water sources [20-22]. Studies from Chennai in 2002 and 2014 have reported almost similar prevalence of intestinal parasites of 60-91% and 75.7%, respectively. *A. lumbricoides* was the predominant helminth, followed by *T. trichiura* in both studies at 52.8% and 6.2%, respectively. In our study, the most common protozoan parasite found among positive monoinfected respondents was *E. histolytica/E.* *dispar*, followed by *G. intestinalis*, and among the helminths, *H. nana* was the most common parasite causing monoinfection, with *Strongyloides stercoralis* as the second commonest helminth causing monoinfection. In 2014, Dhanabal et al. reported that Saidapet and Thiruvanmiyur had the maximum positive cases in Chennai, which was associated with poor quality and unmaintained crude toilets. *Entamoeba coli* infection was present in 23%, followed by 22% *Cyclospora* infections. Among pathogenic parasites, *E. histolytica *(21.8%) and *G. intestinalis* (14.4%) positive infections were documented, with metazoal infection of *H. nana* being predominant at 2.7%. Their study recommended personal hygiene and regular medical examination in low socioeconomic areas [23,24]. Kattula et al. documented that living in a field hut was an important risk factor for acquiring soil-transmitted helminth infection among children. In rural south India, people residing in huts in fields far away from the main village are socioeconomically deprived, and children walk barefoot through “fecal fields” that surround the village because OFD is a common practice [25]. In their study, Kattula et al. documented the following univariate analysis that compared to non-agricultural groups, farm workers were more susceptible to acquiring *Ancylostoma duodenale* infections, with OR of 1.68 and 95% CI of 1.31-2.17 (p < 0.001). Among farm workers with *A. duodenale* infections, duration of work of three to four days and > four days had OR of 1.37, 95% CI of 0.94-1.99, and p-value of 0.098 and OR of 1.61, 95% CI of 1.04-2.47, and p-value of 0.032, respectively, when compared to farm work of one to two days per week. Variables such as personal hygiene, hand washing, OFD, pig rearing, and domestic animal exposure were not significantly associated with *Ancylostoma duodenale* infection. Non-compliance to hand washing with soap and water has an OR of 1.84, 95% CI of 1.27-2.67, and p-value of 0.001 for *Ancylostoma duodenale* infection, which was a statistically significant post adjustment with other variables in a multivariate analysis [26]. This finding was very similar to our finding of only handwashing as the independent predictor having an OR of 3.6 with a CI of 1.00-13.00. Kotian et al. also had similar findings and showed that intestinal parasitic infection was directly related to poor personal hygiene, poor socioeconomic conditions, and other factors. It has been shown that most endemic transmission of enteric infections among communities in developing countries is not primarily via water but instead through other routes, such as contaminated food, hands, and clothing. In their study, it was observed that the prevalence of intestinal parasitic infection was seen more among females (17.07%) than males (8.33%), which was explained as women in their area were engaged in the handling of livestock and doing fieldwork too, other than household work [26]. On February 10, 2015, in the Indian state of Madhya Pradesh, National Deworming Day (NDD) was organized across 51 districts. Of the population between one and 19 years old, of nearly 2.08 crore that was aimed at, approximately 1.84 crore received the benefit with 89% coverage [27]. None of our study participants' families were aware of the deworming program being held by the Madhya Pradesh state nor had they received the benefit of the same due to their nomadic lifestyle.

## Conclusions

Screening for intestinal parasitic infections among migrant construction workers and their households is very important due to their high prevalence among this group. Deworming as a prevention and awareness program is not completely reaching this group. In addition, with a higher prevalence of protozoan parasites, primary prevention strategies, such as education, safe drinking water, proper use of toilets, use of footwear, and above all, awareness to perform proper hand washing with soap and water, for migrant workers and their families need more reinforcement in their workplaces.
